# Rationale and design of a multi-centre randomised controlled trial of individualised cooled dialysate to prevent left ventricular systolic dysfunction in haemodialysis patients

**DOI:** 10.1186/1471-2369-13-45

**Published:** 2012-06-21

**Authors:** Aghogho Odudu, Mohamed Tarek Eldehni, Apostolos Fakis, Christopher William McIntyre

**Affiliations:** 1 Department of Renal Medicine, Derby Hospitals NHS Foundation Trust, Derby, UK; 2 Graduate Entry Medical School, University Of Nottingham, Nottingham, UK; 3 Medical Statistician, Derby Hospitals NHS Foundation Trust, Derby, UK

**Keywords:** Magnetic resonance imaging, Dialysate, Haemodialysis, Left ventricular dysfunction, Randomised controlled trial

## Abstract

**Background:**

The main hypothesis of this study is that patients having regular conventional haemodialysis (HD) will have a smaller decline in cardiac systolic function by using cooler dialysate. Cooler dialysate may also be beneficial for brain function.

**Methods/Design:**

The trial is a multicentre, prospective, randomised, un-blinded, controlled trial. Patients will be randomised 1:1 to use a dialysate temperature of 37°C for 12 months or an individualised cooled dialysate. The latter will be set at 0.5°C less than the patient’s own temperature, determined from the mean of 6 prior treatment sessions with a tympanic thermometer, up to a maximum of 36°C. Protocol adherence will be regularly checked. Inclusion criteria are incident adult HD patients within 180 days of commencing in-centre treatment 3 times per week with capacity to consent for the trial and without contra-indications for magnetic resonance imaging. Exclusion criteria include not meeting inclusion criteria, inability to tolerate magnetic resonance imaging and New York Heart Association Grade IV heart failure. During the study period, resting cardiac and cerebral magnetic resonance imaging will be performed at baseline and 12 months on an inter-dialytic day. Cardiovascular performance during HD will also be assessed by continuous cardiac output monitors, intra-dialytic echocardiography and biomarkers at baseline and 12 months. The primary outcome measure is a 5% between-group difference in left ventricular ejection fraction measured by cardiac magnetic resonance imaging at 12 months compared to baseline. Analysis will be by intention-to-treat. Secondary outcome measures will include changes in cerebral microstructure and changes in cardiovascular performance during HD. A total of 73 patients have been recruited into the trial from four UK centres. The trial is funded by a Research for Patient Benefit Grant from the National Institute of Healthcare Research. AO is funded by a British Heart Foundation Clinical Research Training Fellowship Grant. The funders had no role in the design of the study.

**Discussion:**

This investigator-initiated study has been designed to provide evidence to help nephrologists determine the optimal dialysate temperature for preserving cardiac and cerebral function in HD patients.

**Trial registration:**

ISRCTN00206012 and UKCRN ID 7422

## Background

It is well recognised that HD patients suffer excess cardiac morbidity and mortality and this is mainly due to cardiac failure and sudden cardiac death. Interventions of proven benefit in the non-HD population have thus far been ineffective in HD patients and there is a need for a novel approach.

### Haemodialysis induced myocardial ischaemia

In the setting of coronary artery disease without kidney disease, transient myocardial ischaemia may lead to left ventricular dysfunction that can persist for several hours after the return of normal perfusion, but is eventually followed by functional recovery. This reversible post-ischaemic dysfunction is known as myocardial stunning. Repetitive episodes of stunning are cumulative eventually leading to hibernation then irreversible contractile dysfunction [1]. The ability of HD to induce myocardial ischaemia had been long suspected with reports of silent, reversible ischaemic ECG changes during HD older than twenty years. Other studies showed elevated cardiac troponins were common and up to 30% of HD treatments were complicated by significant intra-dialytic hypotension [2]. These data suggested HD might induce myocardial ischaemia but were unable to prove this or elucidate the pathophysiology. Subsequent proof-of-concept studies by our group and others in subjects with normal coronary arteries have demonstrated that HD is characterised by a 30% reduction in regional myocardial blood flow by H_2_^15^O positron emission tomography [3]. The decreased myocardial blood flow localises to the same segments as regional wall motion abnormalities (RWMAs) using simultaneous 2D-echocardiography at peak-stress, 15 minutes before the end of a HD treatment compared to pre-HD [3]. This supported use of these stress-RWMAs as a practical surrogate for myocardial ischaemia. These changes in the absence of coronary artery disease can be explained by decreased coronary flow reserve which is well described in dialysis populations [4] and is in turn promoted by a high prevalence of autonomic dysfunction, endothelial dysfunction, left ventricular hypertrophy and arterial stiffness. These factors may underlie the reduced sensitivity and specificity of ischaemia stress testing in HD patients [5]. The HD procedure involves a rise in core temperature, extracorporeal circuit, fluid removal and exposure to a dialysis membrane which combine to form a haemodynamic stress provoking demand ischaemia [6]. Our natural history study of 70 prevalent HD patients showed that compared to pre-HD echocardiography, new RWMAs developed at peak-stress in 45 participants and this associated with 30% mortality and 13% reduction in mean left ventricular ejection fraction by 1 year [7,8]. RWMAs persisted at follow-up in the 36/45 survivors of the subgroup. In the cohort who had no abnormal segments at the start of the study, new segmental RWMAs had also developed. Multivariable analysis showed the significant determinants of these stress-RWMAs were age, ultrafiltration volume, intra-dialytic hypotension and serum troponin-T concentration and this displaced all other variables including diabetes status and history of ischaemic heart disease [7]. In short, the RWMAs characterise those who suffer ischaemia due to the haemodynamic stress of HD. Regardless of the balance of micro- and macrovascular disease in the pathophysiology, reducing these RWMAs represents a valid target and may lead to detectable amelioration of global ventricular function.

### Rationale for cooling

Extending the frequency and length of dialysis to reduce ultrafiltration volumes and rates might be one approach to improve cardiac outcomes but the access to and acceptability of this strategy might be limited and unfeasible in some healthcare systems [9]. Novel approaches to reduce RWMAs within the constraints of a standard 4 hour treatment and without the need for invasive tests are needed to retard the development of irreversible cardiac dysfunction and reduced survival. Our pilot studies have demonstrated that cooling the dialysate used during HD reduces the number of episodes of intra-dialytic hypotension and the proportion of patients who develop RWMAs [10-12]. The mechanisms by which lower dialysate temperature may improve haemodynamic instability include reducing heat transfer from the dialysate which contributes to dilation of thermoregulatory blood vessels [13,14]. Cooling the dialysate is not a new intervention [15]. However, prior studies have used prevalent patients, not been randomised or lasted less than one month focussing on short-term benefits of preventing intra-dialytic hypotension. They have also lacked statistical power to detect outcomes validated to predict development of heart failure, mortality and hospitalisation. This study is novel in that the population is incident, the intervention is applied for one year and it is designed to detect long-term changes of validated surrogate outcomes. There are many strategies employed in cooling the dialysate and we previously conducted a systematic review [16]. These include:

 1. Fixed temperature reduction

 2. Biofeedback systems which vary dialysate temperature to deliver either;

 a. no overall change in the patient’s blood temperature (isothermic dialysis)

 b. lowering of the patient’s blood temperature (hypothermic dialysis)

 3. Sequential fixed temperature reduction to a minimum of 35°C or the minimum tolerated above 35°C.

We elected for cooling the dialysate to 0.5°C less than the patient’s temperature (determined with a tympanic thermometer) as a pilot study of 11 patients showed this might achieve the same benefit without the symptoms of cold intolerance which may have been a factor in preventing more widespread use of this strategy [12]. Biofeedback dialysis is a potentially attractive option but has the major disadvantage of requiring specialised dialysis machines which are not universally available. Previous pilot data in short-term studies demonstrated the potential benefit in terms of reduced RWMAs of cooler dialysate which is available on all dialysis machines and can be implemented with minimal or no financial impact.

### Plausibility of HD induced cerebral ischemia

It is increasingly recognised that HD patients exhibit higher levels of functional and cognitive deficits. Dementia is highly prevalent in the HD population with a reported 7-fold relative risk of developing multi-infarct dementia within a year of commencing dialysis compared to age-matched controls; delirium and depression were also found to be common in this population when followed for a period of 4 years [17,18]. Furthermore, even HD patients with normal dementia scores develop neurocognitive dysfunction affecting retention of complex verbal information, memory, executive function, language and mental processing speed [19,20]. Few studies have linked cognitive impairment with findings on brain imaging. Cerebral atrophy is a common finding in HD patients [18,21]. However, the predominant morphological abnormalities reported are white matter hyperintensities termed leukoaraiosis. These areas of high signal on T2-weighted MRI represent white matter ischemia characterised by neuronal loss and demyelination. Although leukoaraiosis develops with aging as a reflection of the burden of cardiovascular disease, many studies report a greater volume of leukoaraiosis in dialysis patients compared with age-matched controls [22,23]. A recent meta-analysis showed leukoaraiosis was associated with a higher risk of stroke, dementia and death in the general population [24]. Adequate perfusion of brain white matter depends on blood pressure and the brain’s autoregulatory capacity [25]. HD can induce haemodynamic and circulatory stress and HD patients have impaired autonomic function and autoregulatory capacity rendering them vulnerable to those haemodynamic changes [26]. Hence the HD process might play an important role in the pathogenesis of brain injury in HD patients.

### Hypothesis

We propose a study to determine whether cooling the dialysate retards the longer term development of cardiac systolic dysfunction. This study will also examine whether cooling the dialysate will have an abrogating effect on cerebral ischaemia and a wide range of functional measures including haemodynamics, microvascular function and validated measures of cognitive impairment. This work has the potential to reveal a refined yet pragmatic intervention to combat excess cardiovascular disease in HD patients.

## Methods/Design

Ethical approval for trial has been obtained from Nottingham Ethics Committee for all participating centres prior to study initiation and patient enrolment. The study will be performed in accordance with the Research Governance Framework, International Conference on Harmonisation Good Clinical Practice Guideline and the 2000 Scotland Revision of the Declaration of Helsinki. All participants are to provide written informed consent before any trial related procedure can occur.

### Participants

Patients must fulfil the following eligibility criteria to be considered for the study enrolment or participation:

Inclusion criteria:

 1. Patients having in-centre HD at least 3 times per week.

 2. Willing and able to provide consent.

 3. Male and female, age ≥ 16 years old.

Exclusion criteria:

 1. Exposure to haemodialysis for >180 days

 2. Contraindications for using MRI (eg. pacemakers and metal implants, pregnancy or lactating)

 3. Inability to tolerate MRI due to claustrophobia

 4. New York Heart Association grade IV heart failure

 5. Mental incapacity to consent

### Study design

**Figure 1:**
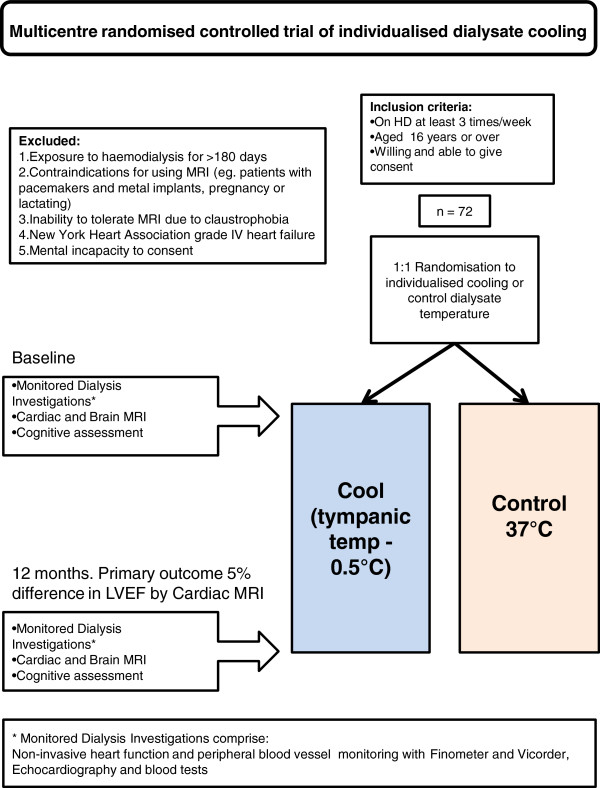
Trial design.

The schedule of events is summarized in Figure 1. The trial is a multicentre, prospective, randomised, un-blinded, controlled trial. Patients will be recruited from 4 research sites and randomised 1:1 to the control group or intervention group. Randomisation will be in a single block by sealed envelopes generated by an independent statistician. The control group will use a dialysate temperature of 37°C for 12 months. The intervention group will have an individualised cooled dialysate temperature for 12 months. This will be set at 0.5°C less than the patient’s own temperature, determined from the mean of 6 prior treatment sessions with a tympanic thermometer, up to a maximum of 36°C, ensuring a minimum temperature separation of 1°C between groups. Adherence to the allocated dialysate temperature will be regularly checked and recorded by nursing staff at each research site enabling distinction between the prescribed and delivered dialysate temperatures. Analysis will be by intention-to-treat regardless of any crossover between delivered temperatures.

### Blinding

This is an un-blinded study. Therefore, after randomisation, the investigators, HD unit staff and patient will be aware of group allocation. This is necessary to ensure adherence to the study protocol. There will be blinding of treatment allocation for analysis of cardiac images for the primary outcome.

### Concurrent treatments

Patients in each trial arm will undergo standard management, as per local HD unit protocols with the exception of dialysate temperature.

### Cardiac and cerebral magnetic resonance imaging

Contrast-free cardiac and cerebral MRI will be performed at baseline and 12 months when the patient has been established on HD with a stable dry weight unchanged for at least 4 weeks. Studies will occur on non-dialysis days at two centres using a 1.5 T MRI scanner (GE Signa HDxt 1.5 T, GE Healthcare, Milwaukee,US). Acquisition will include serial contiguous short-axis cines piloted from vertical or horizontal long axis of the left ventricle using ECG-gated steady-state in free precession sequences in accordance with validated methodologies [27]. Analysis will be performed with the anonymised images off-line by planimetry using commercially available software for assessment of ventricular volumes and mass. Brain MRI will examine the integrity and microstructure of white matter using diffusion tensor imaging. The anonymised images will be analysed off-line for diffusivity and anisotropy corrected for brain volume.

### Cognitive assessment

Cognitive assessment will be performed on the same day as the MRI scans at baseline and 12 months. The Montreal Cognitive Assessment is a validated tool for the detection of mild cognitive impairment with higher sensitivity and specificity than the Folstein Mini-Mental State Examination for the detection of mild cognitive impairment [28]. Trail Making Tests A and B will be used to examine attention and mental flexibility.

### Assessing cardiovascular performance during HD

Detailed characterisation of cardiovascular performance will be assessed during HD at baseline and 12 months. This occurs during the patient’s usual HD session in their own centre using the same portable data collecting instruments across all centres. 2-dimensional echocardiography will be performed using commercially available equipment (1.5-4Mhz probe, Vivid-I, GE Healthcare) by one of two trained technicians in the left lateral position. Sector width, frequency and depth will be adjusted to produce optimal border definition and a frame-rate of 50–80 per second. Standard apical 2 and 4-chamber image loops will be recorded pre-dialysis (rest) and 15 minutes before the end of dialysis (stress). Image loops will be anonymised and analysed off-line in random order for regional deformation using dedicated software (EchoPac, GE Vingmed). Continuous non-invasive cardiac output monitoring will be recorded using a thoracic bioreactance monitor (NICOM™, Cheetah Medical). This uses phase changes in alternating electrical current to derive cardiac output. This endows a greater precision and signal-to-noise ratio than impedance cardiography. The monitor is validated in both healthy and co-morbid adults [29]. The Finometer™ (TNO Instruments) utilises a finger-cuff to detect beat-to-beat changes in digital arterial pressure and calibrates this against oscillometric brachial artery pressure. The Finometer™ is validated in critical care and HD settings [30].

### Arterial stiffness and body composition

Arterial stiffness measured by pulse wave velocity is independently associated with increased risk of cardiovascular disease in HD patients [31]. Carotid-Femoral pulsewave velocity will be measured by a volume displacement technique [32]. Pre-HD body composition and hydration status will be estimated by segmental multi-frequency bioimpedance [33].

### Primary study outcome

Change in resting LVEF by cardiac magnetic resonance (CMR) at 12 months compared to baseline between the intervention and control group.

### Secondary study outcomes

 · Left ventricular mass by CMR

 · Regional myocardial deformation by speckle-tracking echocardiography and CMR tagging.

 · A range of haemodynamic variables, including cardiac output, pulse rate, heart rate and frequency of intra-dialytic hypotension

 · Change in body composition by bioimpedance

 · Change in cerebral diffusion tensor MRI

 · Change in cognitive assessment scores

 · Change in soluble cardiac biomarkers

### Rationale for primary outcome

We selected LVEF measured by CMR as the primary outcome because previous observational study showed mean reduction in LVEF by echocardiography of 13% over 12 months in patients who displayed RWMAs at baseline. RWMAs were abrogated in the short-term by a dialysate cooling strategy in a separate cohort [10,12]. We also selected LVEF as an objective measure which is predictive of mortality and hospitalisations [34]. A change in LVEF greater than 5% has been shown to be an important cut-off approaching the minimum clinically important difference [35,36]. CMR is the gold-standard for measurement of left ventricular mass and volumes, allowing determination of the primary outcome to a much higher precision compared to alternative methods such as echocardiography. This markedly reduces the sample size required to detect between-group differences in an intervention trial, permitting lower costs and faster reporting of final results [37,38]. Sample estimation is from a recent reference range study which used the same CMR acquisition and analytic techniques planned for this study [27] and were performed by a medical statistician using commercially available software (Nquery Advisor v6).

### Sample size estimation

The primary aim in this study is to detect a 5% difference in LVEF as measured by CMR between the control and intervention group after 12 months. Using a two sample t test and assuming a mean LVEF of 67% in the control arm and equal Standard Deviation in each group of 6% [27], a study of 64 participants (32 per group) would permit the detection of a 5% difference in LVEF from baseline to 12 months between the intervention and control groups, with 90% power at 5%, 2-sided significance level. Allowing for study attrition and death of 10%, we aim to recruit 72 participants to achieve an evaluable sample of 64 participants.

### Statistical methods

All the continuous variables will be tested for normality using their histograms and normality tests. The primary endpoint will be compared between the two groups at specific time points using the Mann U Whitney or Independent T-test. Repeated measures analysis will be used for testing the difference on primary endpoint between tests across the whole period. Poisson regression analysis will be used for comparing the frequency of intra-dialytic RWMAs between the two groups. Haemodynamic variables and other secondary endpoints will be analysed using repeated measures and Bonferroni’s test for multiple comparisons. Paired tests, Wilcoxon Signed test or Paired T-test, will be used for comparing the primary and secondary endpoints between two time points within each group. An alpha error at 0.05 will be judged as significant. Analysis will be performed using SPSS for Windows, v16 or Stata v10. Statistical analysis will be based on an intention-to-treat principle with all data analysed according to the patient’s original study group allocation regardless of death, crossover or early withdrawal.

### Monitoring for adverse events

The number and proportion of participants who report treatment-emergent adverse events will be summarized for each treatment group. Treatment emergent events include events that start on or after allocation to the study assigned dialysate temperature.

### Trial completion

Commencing in September 2009, trial recruitment of 73 patients was completed by January 2012. Follow-up will be completed in January 2013 with results analysed and reported in mid-late 2013. Based on the final number recruited (73), the study has 90% power to detect a 5% difference in LVEF.

## Discussion

This study has been designed to provide evidence to help nephrologists determine the optimal dialysate temperature for preserving cardiac function in HD patients. The cooling protocol has been refined to optimise tolerability as informed by a significant body of preparatory studies to directly impact on HD-induced circulatory stress. Since the study was conceived we have expanded the list of vascular beds potentially vulnerable to this insult to include the kidney, gut and brain [39,40]. This study incorporates the ability to explore the hypothesis that cooler dialysate may be beneficial for brain microstructure and cognitive function.

One of the weaknesses of previous studies in HD patients has been the use of prevalent patients well established on dialysis. This brings a significant ‘survivor bias’ effect, as well as the loss of patients with a less severe risk phenotype over time to transplantation. Although it has made recruitment to this study more challenging we elected to restrict recruitment to patients within 180 days of dialysis initiation.

This study is predicated on measures of cardiac structure and function which are validated surrogate outcomes of relevant patient outcomes. The study design includes a variety of explanatory outcomes which may generate insights into mechanisms of any reported benefits and multi-organ effects of HD treatment. It is conceived that this study may provide the justification and sample size estimation required for a larger study predicated on cardiovascular events and mortality. The plausibility of this approach has been strengthened by recently published observations of reduced cardiovascular mortality in patients dialysed against cooled dialysate [41].

## Conclusion

Dialysate cooling is a well-tolerated, low-cost and universally available intervention. We hope that this pragmatic trial of cooling will provide important data informing the optimal therapy for HD patients. Although some consequences of HD induced circulatory stress might be addressed by extended treatment schedules, thrice weekly, in-centre HD is likely to remain the norm for the majority of HD patients for the foreseeable future. Therefore there remains a need for therapy refinement within that paradigm.

## Abbreviations

LVEF, Left ventricular ejection fraction; CMR, Cardiac magnetic resonance; MRI, Magnetic resonance imaging; RWMAs, Regional wall motion abnormalities; ECG, Electrocardiogram.

## Competing interests

The authors declare no competing interests.

## Authors' contributions

CWM is the principal investigator and conceived the study. CWM, AO, MTE and AF contributed to study design. AF performed sample size estimations and the randomisation. AO, MTE and CWM wrote the manuscript. All authors read and approved the final manuscript.

## Funding

The trial is funded by a Research for Patient Benefit Grant from the UK National Institute of Healthcare Research (Grant ref: PB-PG-0408-16195). AO is funded by a British Heart Foundation Research Training Fellowship Grant (Ref: FS/11/10/28564) with CWM as principal supervisor.

## Pre-publication history

The pre-publication history for this paper can be accessed here:

http://www.biomedcentral.com/1471-2369/13/45/prepub
